# Supplementary material for: Identification of photoperiod-induced specific miRNAs in the adrenal glands of Sunite sheep (Ovis aries)
Source: Front Vet Sci. 2022 Jul 22;9:888207. doi: 10.3389/fvets.2022.888207 (PMC9354845; doi:10.3389/fvets.2022.888207)
Supplement: Supplementary file 1 [file Data_Sheet_1.ZIP › Supplementary file/miRDeep2 file.zip!/miRDeep2 file/Novel_Structure/SP42A2/Novel_88.pdf]

Provisional ID	:	NC_019471.2_27923
Score total	:	18.6
Score for star read(s)	:	3.9
Score for read counts	:	11.3
Score for mfe	:	1.8
Score for randfold	:	1.6
Score for cons. seed	:	
Total read count	:	34
Mature read count	:	31
Loop read count	:	0
Star read count	:	3

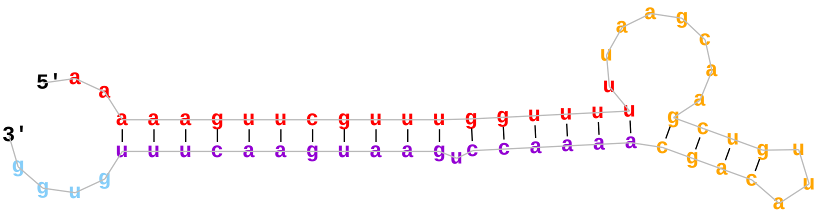

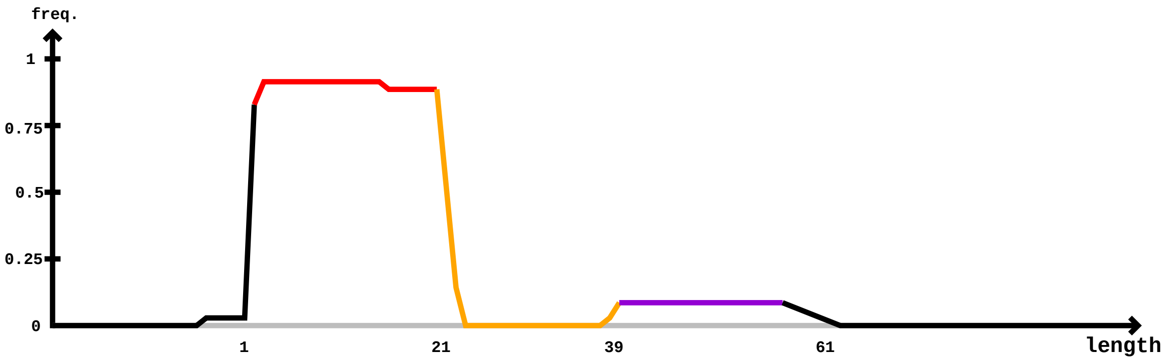

Mature Star

```
5'- auacacuacugggcuggccaaaaaguucguuugguuuuuuaagcaagcuguuacagcaaaaccugaaugaacuuuguggccaacccaagacucuguucccacaggcagaca   -3'    obs
    auacacuacugggcuggccaaaaaguucguuugguuuuuuaagcaagcuguuacagcaaaaccugaaugaacuuuguggccaacccaagacucuguucccacaggcagaca          exp
    .........((((.((((((.(((((((((((((((((........((((...)))))))))).))))))))))).)))))).))))....((((((......))))))..  reads    mm    sample
    ..............uggccaaaaaguucguuug..............................................................................  1        0     seq
    ...................aaaaaguucguuugguuuuu........................................................................  2        0     seq
    ...................aaaaaguucguuuggGuuuu........................................................................  23       1     seq
    ...................aaaaaguucguuugguuuuuu.......................................................................  1        0     seq
    ...................aaaaaguucguuuggGuuuuua......................................................................  2        1     seq
    ....................aaaaguucguuuggGuuuuua......................................................................  3        1     seq
    ........................................................Uaaaaccugaaugaacuuu....................................  1        1     seq
    .........................................................aaaaccAgaaugaacuuu....................................  2        1     seq
```
